# Darwin and Fisher meet at biotech: on the potential of computational molecular evolution in industry

**DOI:** 10.1186/s12862-015-0352-y

**Published:** 2015-05-01

**Authors:** Maria Anisimova

**Affiliations:** Institute of Applied Simulations, School of Life Sciences and Facility Management, Zürich University of Applied Sciences, Einsiedlerstrasse 31a, Wädenswil, 8820 Switzerland; Department of Computer Science, ETH, Zurich, Switzerland; Swiss Institute of Bioinformatics, Lausanne, Switzerland

**Keywords:** Molecular evolution, Applied bioinformatics, Modeling, Selection, Adaptation, Conservation, Drug target, Resistance, Immune response

## Abstract

**Background:**

Today computational molecular evolution is a vibrant research field that benefits from the availability of large and complex new generation sequencing data – ranging from full genomes and proteomes to microbiomes, metabolomes and epigenomes. The grounds for this progress were established long before the discovery of the DNA structure. Specifically, Darwin’s theory of evolution by means of natural selection not only remains relevant today, but also provides a solid basis for computational research with a variety of applications. But a long-term progress in biology was ensured by the mathematical sciences, as exemplified by Sir R. Fisher in early 20th century. Now this is true more than ever: The data size and its complexity require biologists to work in close collaboration with experts in computational sciences, modeling and statistics.

**Results:**

Natural selection drives function conservation and adaptation to emerging pathogens or new environments; selection plays key role in immune and resistance systems. Here I focus on computational methods for evaluating selection in molecular sequences, and argue that they have a high potential for applications. Pharma and biotech industries can successfully use this potential, and should take the initiative to enhance their research and development with state of the art bioinformatics approaches.

**Conclusions:**

This review provides a quick guide to the current computational approaches that apply the evolutionary principles of natural selection to real life problems – from drug target validation, vaccine design and protein engineering to applications in agriculture, ecology and conservation.

## Introduction

For over a century computational scientists have been working side by side with empirical scientists, supporting key developments in molecular and evolutionary biology. Despite this, today close interdisciplinary collaboration can be still somewhat elusive, with different communities of scientists speaking “different languages”. Yet, it is well worth adapting the research process and communication in order to include a wider range of specialists, particularly in industries.

A historical perspective shows that progress in life sciences relies on solid backing from statisticians, mathematicians, computational scientists, and theoreticians in general. Remarkable in this context is the contribution by R. A. Fisher – one of the first bioinformaticians, who developed the statistical theory for experimental design and hypothesis testing, together with many now widely used techniques (eg, the analysis of variance, the method of maximum likelihood, etc.), originally to address the needs of agricultural research at the Rothamsted Experimental Station, Together with S. Wright and J. B. S. Haldane, Fisher has established the field of population genetics, and contributed to the neo-Darwinian evolutionary synthesis, which reconciled Mendelian genetics with Darwin’s evolutionary theory at the level of hereditary molecular information. In the 60s the founders of molecular evolution E. Zuckerkandl and L. Pauling used quantitative comparisons to show that molecular changes in a protein accumulate at a uniform rate [1]. This concept, known as “molecular clock”, enabled the theoretical work by J. Crow and M. Kimura, who modeled genetic drift and selection as realizations of similar processes. The molecular clock served as basis for Kimura’s neutral theory of molecular evolution, whereby selection had no significant influence on shaping genomes with most genetic changes being selectively neutral [2]. The neutral theory greatly contributed to the development of the field as it provided a simple null hypothesis with testable predictions. Since then, numerous statistical tests have been developed and remain highly relevant to detecting selection in genomic data, as emphasized later in this review.

More recently, the availability of high-throughput molecular data served to advance statistical and computational methods for genomics, allowing for a variety of applications – from medical genetics and pharmacology to biotechnology, agriculture and ecology. The size and the complexity of molecular data underline the crucial role of theoreticians and computational scientists for the success of biological data exploration . Molecular data size and complexity have surpassed the so-called “Excel barrier”, so that companies analyzing genomics data can no longer rely on old practices. Indeed, pharma and biotechnology companies see an increasing demand for computational scientists with strong skills in mathematical modeling, machine learning, data mining, complex optimization and data representation (e.g., [3]).

## Review

### The importance of selection studies at the genomic level

The field of computational genomics has been growing steadily, attracting more research funding for both academic and applied research in biotech and pharma companies. Here I focus on the potential of computational methods to study how genomic changes occur over time and their impact on phenotype or genetic fitness [4-6]. While Darwin has described how selection may act on a phenotype, he had no knowledge of hereditary mechanisms, and would have been pleased to see how far we have come today in our understanding of selective mechanisms in molecular sequences. Current computational methods can detect genomic regions under selection and help to describe the biological mechanisms generating the observed molecular patterns. Considering this, computational methods provide effective means of narrowing down the space of plausible candidates or hypotheses for further testing. A diversity of biological mechanisms may cause genetic mutations with various fitness effects, leading to a variety of ways natural selection can manifest itself. The central role of selection on molecular sequences has been demonstrated in the adaptation to new environments, the host-pathogen “arms” race, the emergence of competition, the evolution of complexity, and in the morphological and behavioral evolution, for example see Figure 1 of [7]. Natural selection may act on the protein, on the DNA sequence, and even on whole genomic features. Negative or purifying selection conserves the sequence (or other molecular features), while positive selection acts in a diversifying or a directional manner favoring specific changes. Positive selection typically affects molecular regions involved in genetic conflict, and often acts in an episodic manner (i.e., for a limited time). Selection scans became an indispensible component of genomic studies (e.g., [8,9]), since they help to understand the biological constraints and to identify mutational hotspots due to adaptive processes.

**Figure 1:**
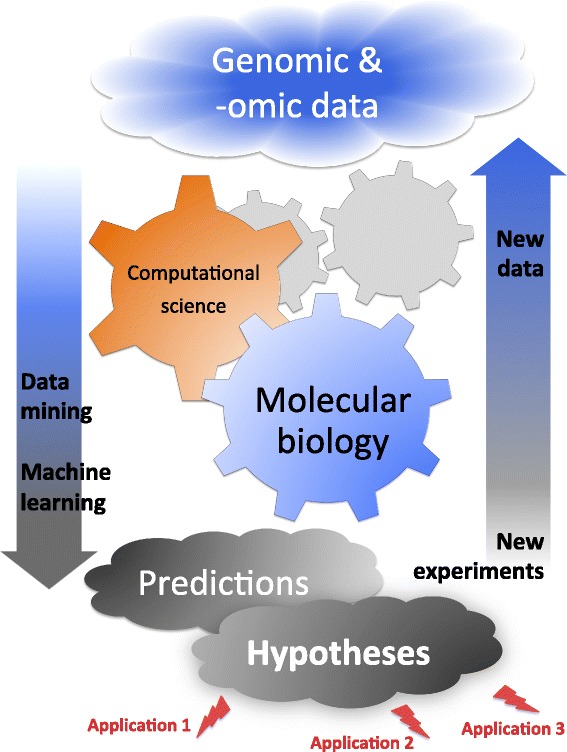
Feedback loop between experimental and computational stages of research and development. Applications of genomics and omics in industry originate from continuous collaborations between theoreticians, computational and experimental scientists in a feedback loop: Computational predictions provide ground for setting up new experiments and generate new data with new levels of complexity. These data are again analyzed by computational scientists to refine the predictions and to generate new hypotheses for further experimental validation. In absence of *a priori* biological hypotheses, exploratory learning approaches can be used to generate new hypotheses or to guide the parametrization choices for new statistical models.

Studies of selective constraints in genomes of populations and species can have a variety of applications (see Table 1 for examples). Identification of deleterious mutations (e.g., mutations causing disease) may aid the development of gene therapies and personalized treatments. Detecting hotspots of diversifying pressure in antigenic sites, epitopes and pathogenic receptors can be used in drug and vaccine design. Phylogenetic methods are increasingly used in immunology and cancer genomics. The analysis of selective pressures and disease transmission rates using host and pathogen samples provides important clues for epidemiology, helping to understand the disease dynamics and to develop predictive strategies for disease control. This applies equally to animal and plant hosts as well as their pathogens, thus having applications also in the domain of agricultural research such as developing molecular-based strategies for increasing crop resistance to pathogens. Similarly, evolutionary studies may provide insights to the genetic basis for stress tolerance and yields of animal and plant products. Other applications of molecular evolution and selection analyses may include biodiversity, conservation, sustainable development, bioremediation, bioengineering and nutrition. Below I briefly draw attention to some successful approaches for studying the evolutionary dynamics in molecular sequences, illustrated by examples.

**Table 1:** **Selected examples of applications of molecular evolution and selection studies**

**Application type**	**Description**	**Citation**	**Computational approach**
**Control of HIV infection**	Protein function study of HIV restriction properties in TRIM5α	[40]	Codon model tests for selection
**Model species selection for pharmaceutical discovery**	Assessment of pharmacological target homology	[42]	Phylogenetic analyses of gene families
**HIV vaccine development**	Assessment of phylogenetic diversity in viral proteins and antibodies; identification of conserved epitopes	[50,53]	Phylogenetic analyses and codon model tests for selection
**Flu epidemics prediction; vaccine strain selection**	Modeling of antigenic dynamics of flu over time	[54]	Phylogenetic diffusion model of antigenic evolution
**Prediction of HIV progression**	Monitoring the synonymous substitution rates in viral protein samples from HIV-positive patients over time	[67]	“Relaxed-clock” modeling of codon evolution
**Evaluating epidemics dynamics and the effect of public health interventions**	Estimating the rates of transmission, recovery, sampling, and the effective reproductive number	[81-83]	Birth-death phylogenetic models
**Flu epidemics prediction; vaccine strain selection**	Modeling adaptive epitope changes and deleterious mutations outside the epitopes in flu from one year to the next	[93]	Molecular evolution modeling over viral genealogies
**Crop resistance**	Identifying the resistant variants of the *Pi-ta* gene in rice that is used to control rice blast disease	[96]	Analyses of genetic diversity and evolution
**Mapping disease associations; complex disease biology; development personalized medicine**	Genome studies identifying sites of genomic diversification, associations with diseases, estimating fitness of mutations	[73,74]	Evolutionary analyses of genomic constraints, genome-wide association studies
*Disease biology; identification of vaccine targets	Population genomics of the sexually transmitted bacteria *Chlamydia trachomatis*	[97]	Genome-wide evolutionary analyses of conservation by codon models and population genetics approaches
*Disease biology	Adaptation in the cavity causing bacteria *Streptococcus mutans*	[98]	Genome-wide evolutionary analyses of conservation and demography
*Conservation and biodiversity; climate change	Evaluating hybridization of blue whale subspecies in southern hemisphere	[99]	Population genetics analyses
*Impact of climate change	Evaluating the interplay between global climate change, genetic diversity and species interactions and community structure	[100]	Evaluation of intraspecific genetic diversity by population genetics approaches

*Highlighted in the 2013 editorial “Highlights in applied evolutionary biology” in the peer-reviewed journal “Evolutionary Applications”.

### Computational approaches to study evolution and selection in molecular sequences

Evaluating selective pressures on molecular sequences relies on the comparative evolutionary approach, and therefore requires at least two homologous sequences [10]. Simple studies of sequence conservation already go towards this objective – they allow to pinpoint functionally important parts of a sequence, based on our understanding of how natural selection acts on molecular sequences. In practice, studies of sequence homology and conservation have been fundamental to the discovery in genomics. In pharma industry, alignment and similarity searches are routinely used together with template-based structure prediction in structure-based drug design (integrated in purpose-built software).

For protein-coding genes, codon models of substitution provide means to expand inferences from simple sequence conservation to more sophisticated modeling of codon substitution through time driven by selection and mutation [11-17]. In such models selection is modeled explicitly, allowing for variation of selection pressure across sequence sites and over time. The power of the approach depends on the number and the range of sequences analyzed [18]. For large samples from well-designed experiments, it is possible to accurately predict the positions and the time episodes where selection has operated [19-22]. Other tests for selection are not specific to coding sequences (for review see [7]). Substitution models in general can be used to detect shifts in evolutionary rates or sequence composition [23-25], which are potentially due to adaptive processes. Neutrality tests based on summary statistics allow inferences of selection if no other demographic factors can be invoked to explain the observed data [26,27]. Besides these methods, selection can be detected using Poisson random-field models [28-30], and tests based on linkage disequilibrium, haplotype structure and population differentiation [31-36].

The basic idea behind all tests for selection is to compare the molecular patterns observed in genomic sequences to what could be expected by chance. Significant deviations point to interesting candidate regions, sites or time episodes, and provide excellent hypotheses for further experimental and statistical testing. Different methods use different statistics to make their inferences about selection. Ideally, the null expectation and alternative scenarios can be described by a statistical model. This enables a proper statistical treatment during parameter estimation, evaluation of uncertainty, hypothesis testing and model selection. Model-based approaches, while desirable, should make sure to use models that account for key biological factors and that are sufficiently robust against violations of key assumptions. It is important to be aware, that biological mechanisms that are not included in the model may have a significant impact on the objective of inference. If it is not possible to include a certain biological factor (e.g., population size) in a model, its influence on the parameter of interest (e.g., selection pressure on the protein) can be investigated using separate carefully designed tests.

In this respect, Markov models of character substitution have been particularly successful at inferring selection at individual sites and lineages. Among widely used methods are likelihood ratio tests of codon substitution models, which detect selection on the protein sequence using the comparison of nonsynonymous (amino-acid altering) and synonymous (amino-acid preserving) substitution rates (for review see [37]). If a test is significant, Bayesian prediction is used to identify the selected positions or lineages affected by selection. The pharmaceutical giant GlaxoSmithKline (GSK) acknowledged the applied value of these methods by an award to the principal investigator Prof Ziheng Yang (UCL, UK). The relevance of selection analyses with codon models for downstream applications can be demonstrated on a selection of case studies. A classic example is the human major histocompatibility complex molecules of class I (glycoproteins mediating cellular immunity against intracellular pathogens), where all residues under diversifying selection pressure were found clustered in the antigen recognition site [38,39]. In another example, selection analyses identified a sequence region of 13 amino acids with many positive-selected sites in TRIM5α, involved in cellular antiviral defense [40]. Functional studies of chimeric TRIM5α genes showed that the detected region was responsible for the difference in function between the rhesus monkey linage where TRIM5α restricts HIV-1 and the human TRIM5α that has only weak restriction.

More generally, the numerous genome-wide scans in mammals agree that genes affected by positive diversifying selection are largely responsible for sensory perception, immunity and defense functions [41]. Consequently, pharma and biotech companies should make a greater use of computational approaches to detect genes and biochemical pathways subject to differential adaptive evolution in human and other lineages used as experimental model organisms, for example as it has been done by R & D of GSK [42,43]. Such studies can be extremely valuable, for example when selecting drug targets. Particularly, evolutionary analyses can pinpoint evolutionary differences between model organisms used for drug target selection. Such differences can be responsible for unpredicted disparities in response to medical treatment, as it has been highlighted by the tragic effects of TGN1412 treatment during human drug trials in 2006 [44]. Selection analyses are also important for research in agriculture or conservation, since in plant genomes positive selection affects most notably disease resistance genes [45,46], defense enzymes such as chitinases [47] and genes responsible for stress tolerance [48]. Consequently evolutionary studies help to detect proteins, binding sites and their interactions relevant for host-pathogen coevolution. For example, diversifying selection drives the evolution of several exposed residues in leucine-rich repeats (LRRs) of the bacterial type III effectors (that attack plant defense system) from the phytopathogenic *R. Solancearum* infecting >200 of plant varieties including agriculturally important crops [49]. Similarly, studies of phylogenetic diversity and selection in viral strains and antibody sequences are contributing to the new HIV vaccine development strategy, whereby antibodies are designed to bind to conserved epitopes of selected viral targets [50-53]. Moreover, molecular evolution modeling approaches can greatly enhance the modeling of antigenic dynamics of pathogens over time (e.g., [54]).

In protein coding sequences selection may also act on the DNA, whereby synonymous codon changes may affect protein’s stability, expression, structure and function [55,56]. Translational selection manifests itself as the overall codon bias in a gene to match the abundances of cognate tRNA. Remarkably, this property can be successfully used in biotechnology, for example to dramatically increase transgene expression by synthesizing sequences with optimal synonymous codons [57]. Optimal codon usage may be approximated by codon usage bias – using bioinformatics methods [58]. Besides this, more subtle selective mechanisms may act on certain codon positions; affecting splicing, mRNA stability, gene regulation protein abundance, folding and function (e.g., [59-63]). In human genes this may lead to disease (such as cancers and diabetes) or may be responsible for differences in individual responses to drug treatment (e.g., [64]). Haplotypes with synonymous changes may have increased fitness and will be consequently increase in frequency in a population. Therefore, the knowledge of these specific synonymous polymorphisms may be important to explain differential treatment effects in population and contribute to the development of personalized medicines [65]. Molecular evolution methods are powerful enough to detect such interesting candidate cases: Recent study of synonymous rates detected many disease related genes, particularly associated with various cancers, as well as many metabolizing enzymes and transporters, which affect the disposition, safety and efficacy of small molecule drugs in pharmacogenetics [66]. This shows that computational molecular evolution studies have real power to predict genes and codon positions where a replacement of synonymous codons changes protein fitness. Such predictions promise to be valuable for applications in protein engineering. Indeed, some biotech companies such as DAPCEL are already using the knowledge of interesting synonymous positions for enhanced protein production. Compared to laborious and time-consuming trial-and error experiments, computational prediction offers a fast way of obtaining candidate genes and positions for experimental validation. Furthermore, monitoring of the synonymous rates may be also informative for diagnostics purposes, as has been shown in evolutionary studies of serial viral samples from HIV-positive patients [67].

Species evolution is however a result of complex population dynamics, making population scale studies of genetic diversity a powerful complement to codon-based selection analyses. Successful population level techniques include tests of neutrality [26], Poisson random-field models (e.g., [68,69]) combined with demographic modeling and genome-wide association studies [70]. These methods apply to full genome sequences helping to identify also non-coding genomic regions of functional relevance and those associated with certain population traits. For medical genetics, uncovering the relevance of genomic variation in populations helps to pinpoint the disease variants and use this information in the development of personalized medicines and treatments. Determining fitness of specific mutations is now possible using macro-evolutionary inferences and population genetics approaches [71-73], which can be successfully combined with genome-wide association studies [74]. These inferences could be combined with applications in a clinical context [75].

However, many traits are shaped by multiple loci so that the effects of any single mutation can be observed only through their epistatic effects [76,77]. Consequently, computational approaches recently extended single loci inferences to detecting epistatic effects of mutations through the identification of polygenic selection, i.e., whereby selection affects whole gene clusters whose protein products interconnected in the biological pathways that they share. Such analyses found that polygenic selection often affects pathways involved in immune response and adaptation to pathogens [78], which is also consistent with results from single loci studies.

Another approach for detecting selective signatures is based on detecting shifts in evolutionary substitution rates over time, for example based on covarion or Markov modulated models [16,79]. Such methods may be used to detect functional shifts in proteins of interest, providing evolutionary information that aids structural and functional protein prediction. Therefore such analyses can be helpful for many pharma and biotech applications that use structural modeling to design proteins and peptides for therapeutic or other biotechnology applications (e.g., [80]). Alternatively, changing diversification rates can provide evidence for changing environments, emerging pathogens and shed light on epidemiological dynamics. Diversification bursts or exponential growth, for example, may represent the emergence of particularly virulent strains resulting in epidemics. Such selective signatures can be characterized by phylogenies or genealogies relating the molecular sequences in a viral sample based on the birth-death models of stochastic branching processes [81]. This approach allows to evaluate the effects of public health interventions by estimating the rates of transmission, recovery, and sampling, and consequently, the effective reproductive number. For epidemiology-related problems, these techniques become particularly powerful when combined with classical epidemiologic models SIR or SIS [82,83]. Evolutionary methods can be useful also for the analyses of somatic hypermutation in antibody sequences during antibody maturation, or for monitoring somatic mutations in cancerous tissues [84-87]. Indeed, applications of phylogenetic methods to cancer and immunology research are now attracting more attention and funding (e.g., [88,89]).

Selection may also operate on whole genomic features, such as indels, gene order, gene copy numbers, transposable elements, miRNAs, post-translational modifications, etc. To detect selective signatures of conservation or adaptation, the observed genomic patterns are compared with a neutral expectation, i.e., patterns that can arise by chance alone. For example, phylogenetic patterns produced by tandem repeats in eukaryotic proteins can be used to identify interesting candidate genes that might be under diversifying pressures [90]. In plants a similar analysis strongly pointed to lineages where diversification (in terms of unit number and their order conservation) occurs in LRRs that are found in abundance in plant resistance genes [91]. Such analyses allow for example to pinpoint the relevant genes and lineages where selection on tandem repeat units is due to adaptation to emerging pathogens or to changing environmental conditions. This opens the door to applications such as synthetically introducing identified gene variants into plant genomes to produce crops with improved resistance or better stress tolerance properties. Indeed, crop protection agencies and companies (e.g., Syngenta, Rothamsted Research) have started using evolutionary analyses to elucidate the origins of resistance to pathogens [92].

Even when selection study is not the goal of the analyses, modeling its influence on genomic data is of utmost importance. Failing to do so may lead to biased and inaccurate inferences that could misguide follow-up experimental studies. However, modeling selection enhances the predictive power of methods that are used to study adaptive or antagonistic processes. A nice example is the recent predictive fitness model for influenza, which couples the fitness values and frequencies of strains with molecular evolution modeling on an influenza stain genealogy for haemaglutinin gene [93]. This approach uses observed viral samples taken from year to year to predict evolutionary flu dynamics in the coming year, which is practically relevant for selecting vaccine strains for the new flu season.

## Conclusions

The last decades have seen the development of accurate and powerful computational methods for evaluating evolution and selection in molecular data. Both industry and basic research should discover and exploit the full potential of these methods, as they provide efficient means to generate viable biological hypotheses, including interesting candidates cases for further experimental testing. Examples above included such successful applications. Genomics and omics data provides immense opportunities for applications in industry. While pharma and biotech giants are generally aware of this, employing their own bioinformaticians, smaller companies often do not know of current possibilities provided by the state-of the art computational methods and the volumes of newly generated data. But even bioinformatics teams in pharmaceutical giants usually have no sufficient capacity to develop suitable techniques for the analysis of their data, as they focus on more imminent results for their company. The development of robust statistical methodology demands substantial time doing basic research. Further, even for method developers it is hard to keep pace with all the relevant advances in the field. For this reason, industry should actively engage with researchers in academia – starting with networking and discussions of company’s needs and the potentially useful academic results, gradually bringing this into productive industry-academia collaborations.

Finally, productive collaborations require efficient communication between theoreticians, computational and experimental scientists, with a continued feedback loop built into the research process (Figure 1). While research in biology is traditionally hypothesis-driven, current volumes of new complex data also require exploratory learning approaches. Therefore today, machine learning, pattern recognition and data mining approaches became essential in the exploration of big data. Such techniques can help with the choice of suitable model parameterizations [94]. Computational predictions can be used to formulate new biological hypotheses. To validate these hypotheses, new experiments should be set up in order to generate new data, possibly with new levels of complexity. These data can be analyzed again by computational scientists, in order to refine the initial predictions and to refine or generate new hypotheses for further experimental validation, re-starting the loop (Figure 1). While it is easy to generate genomic data today, greater thought must be invested into the experimental design, in order to make the statistical inferences more accurate and informative. This requires solid expertise in statistics.

To conclude, that pharma and biotech companies should actively seize upon the potential of computational molecular evolution approaches in their translational research. As shown above, this can include drug target identification and validation, animal model selection, preclinical safety assessment, vaccine design, epidemics control and drug repositioning. Such techniques are promising to become mainstream, strengthening the current position of translational research in industry. The translational value of computational molecular evolution is not limited to health and pharma industry, but also include a variety of other exciting applications – protein engineering, agriculture, environmental risk assessment, ecology, biodiversity and conservation. Again, this cannot be done without strong interdisciplinary partnerships. Bioinformatics has now become a vibrant and highly interdisciplinary area of research and the outlook for its future and its applications is very optimistic – “Bioinformatics alive and kicking” [95].
